# Causality between six psychiatric disorders and digestive tract cancers risk: a two-sample Mendelian randomization study

**DOI:** 10.1038/s41598-024-66535-7

**Published:** 2024-07-19

**Authors:** Qi Fu, Linghui Li, Niyang Zhuoma, Rui Ma, Zhixi Zhao, Zhaxi Quzuo, Zhen Wang, Deji Yangzong, Ji Di

**Affiliations:** 1 Qinghai University Affiliated Hospital (The Clinical Medical School), Qinghai University, Xining, 810000 Qinghai China; 2 The Fifth People’s Hospital of Qinghai Province, Xining, 810000 Qinghai China; 3 Yushu City People’s Hospital, Yushu, 815099 Qinghai China

**Keywords:** Colorectal cancer, Esophagus cancer, Gastric cancer, Mendelian randomization, Psychiatric disorders, Cancer, Psychology, Diseases, Oncology

## Abstract

Associations between psychiatric disorders and digestive tract cancers have been proposed. However, the causal link between these factors remains unclear. This study pioneers Mendelian randomization (MR) analysis to explore the genetic link between psychiatric disorders and digestive tract cancers risk. We analysed data on six psychiatric disorders [schizophrenia, bipolar disorder, major depressive disorder (MDD), attention deficit hyperactivity disorder, autism spectrum disorder, and panic disorder (PD)] and digestive tract cancers [esophagus cancer (EC), gastric cancer (GC), and colorectal cancer (CRC)] from genome-wide association studies databases. Using instrumental variables identified from significant single nucleotide polymorphism associations, we employed the inverse variance weighted (IVW) method alongside the weighted median (WM) method and MR-Egger regression. The results revealed no causal link between psychiatric disorders and the risk of EC or GC. Psychiatric disorders were not identified as risk factors for CRC. Notably, PD demonstrated a lower CRC risk (OR = 0.79, 95% CI 0.66–0.93, *P* = 0.01). This MR analysis underscores the lack of a causal association between psychiatric disorders and digestive tract cancers risk while suggesting a potential protective effect of PD against CRC.

## Introduction

Digestive tract cancers predominantly encompass esophagus cancer (EC), gastric cancer (GC), and colorectal cancer (CRC) and are collectively characterised by elevated morbidity and mortality rates^1^. EC is the eighth most prevalent malignancy globally and the sixth leading cause of cancer-related mortality. The number of deaths from esophagus cancer is 500,000 each year^2,3^. The five-year survival rate for EC shows a notable decrease, registering merely 20% in the United States in 2023 and 40.1% in China in 2018^1,4^. Sex, race, smoking, alcohol consumption, dietary habits, and nutritional status are recognized as risk factors for EC^5^. Epidemiological studies in 2022 disclosed GC as the fifth most common cancer globally, with an annual incidence surpassing 1.1 million^6^. Concurrently, GC has a high mortality rate, positioning it as the fourth leading cause of cancer-associated deaths^7^. According to statistical data, GC caused 800,000 deaths worldwide in 2020^8^. Age, the presence of Helicobacter pylori, and reduced consumption of fruits and vegetables have been identified as risk factors for the development of GC^9^. CRC has a high incidence rate. Global cancer data from 2018 showed that 10% of all cancers diagnosed each year were CRC^10^. It is the second most prevalent cancer in men and the third most common in women. The overall number of individuals with CRC is increasing, with a projected 2.5 million new cases anticipated by the year 2035^11^. Risk factors associated with CRC include sex, age, genetic predisposition, and inflammatory processes. Although numerous risk factors associated with digestive tract cancers have been validated, a multitude of potential risk factors, including psychiatric disorders, remain unexplored. Investigating the causal connections between these unidentified risk factors and digestive tract cancers holds clinical promise and offers valuable insights for the prevention and treatment of such malignancies.

The term ‘psychiatric disorder’ describes conditions marked by deviations in perception, behaviour, and cognition. Psychiatric disorders often lead to suicidal behaviour, which is one of the most important public health problems. Dysregulation of the kynurenine pathway, resulting in an imbalance in neuroactive metabolites, has been suggested as a biomarker for both psychiatric disorders and suicidal behaviour^12^. Psychiatric disorders include schizophrenia, bipolar disorder (BD), major depressive disorder (MDD), attention deficit hyperactivity disorder (ADHD), autism spectrum disorder (ASD), and panic disorder (PD)^13^. Schizophrenia is characterised by hallucinations, cognitive impairment, and diminished social capacity. A survey conducted in 2020 showed that the lifetime prevalence in the American population was 1%^14^. It has been substantiated as a risk factor for various conditions, such as cardiovascular diseases and is emerging as a focal point in research on the occurrence and progression of digestive tract cancers^15^. However, conclusive evidence establishing a direct association between schizophrenia and digestive tract cancers is currently lacking. BD is categorised into types I and II, characterised by the predominant presence of manic episodes in the former and depressive episodes in the latter, with the potential for alternation between the two manifestations^16^. A 2022 study showed that the lifetime prevalence of bipolar disorder in the American population was 2.4%^17^. Previous studies have reported an elevated risk of cancer, notably breast cancer, in individuals diagnosed with BD. However, whether BD serves as a risk factor for digestive tract cancers warrants further exploration^18^. MDD is characterised by enduring negative emotional states, diminished interest in both the occupational and personal spheres and a recurrent inclination toward suicidal ideation^19^. In 2020, the incidence of severe depression in Australia was 4.6%, posing a major threat to life and health^20^. Recent studies have shown a frequent link between vulnerability to depression, psychological distress, hopelessness, and demoralisation. Demoralisation, alone or in combination with depression, has been identified as a major risk factor for suicidal ideation and suicidal behaviour^21^. Recently, an increasing number of researchers have focused on elucidating the association between major depression and cancer. According to a meta-analysis, MDD does not confer an increased risk of developing cancer^22^. A Mendelian randomization (MR) study suggested that MDD is a potential risk factor for breast cancer^23^. Conversely, another MR study revealed no discernible causal relationship between MDD and prostate cancer^24^. However, the precise interplay between MDD and digestive tract cancers remains unclear. ADHD manifests predominantly in the paediatric population, with an incidence of 3.4% in Europe in 2016^25^. Characterised by traits, such as distractibility, impaired concentration, heightened motor activity, and impulsivity, these symptoms frequently persist throughout adulthood^26^. A cohort investigation posited heightened susceptibility to CRC in individuals diagnosed with ADHD^27^. However, few studies have focused on the correlation between ADHD and malignancies of the esophagus and stomach. The endeavour to substantiate the association between ADHD and neoplastic occurrences within the digestive tract through rigorous scientific methodologies represents an innovative research paradigm. ASD is a neurodevelopmental disorder characterised by impairments in social communication and repetitive behavioural patterns, manifesting in 2.3% of the paediatric population and 2.2% of the adult population within the United States^28^. In a cohort study, no evidence was found to establish an association between ASD and increased susceptibility to cancer risk^29^. Nonetheless, some scholars argue that a pleiotropic link exists between ASD and cancer risk^30^. The association between autism and tumours, particularly digestive tract cancers, remains controversial across various studies. Further research is required to conclusively validate or refute these relationships. PD is a prevalent psychiatric condition characterised by fear, panic episodes, and avoidance behaviours^31^. The interplay between PD and cancer risk has not been thoroughly explored, with a recent MR analysis indicating that PD is not a contributing risk factor for breast cancer^32^. However, PD as a potential risk factor for digestive tract cancers deserves attention as an area for further investigation.

Mental illnesses are closely related to neurodegenerative diseases and can lead to nerve cell death and neurodegeneration^33^. Cancer is primarily caused by the uncontrolled proliferation of cells^34^. There is evidence that common biological mechanisms of these two diseases, such as oxidative stress, metabolic disorders, and inflammation, promote not only apoptosis but also cell proliferation^35^. Some immune metabolic markers observed in patients with psychiatric disorders may also be associated with the development of cancers^36^.

Prior investigations have suggested a connection between specific psychiatric disorders and digestive tract cancers. However, the strength of the causal link remains uncertain due to potential biases, such as reverse causation and confounding factors. Mendelian randomization (MR) analysis, which employs single nucleotide polymorphisms (SNPs) as instrumental variables (IVs), has emerged as a method for inferring causal associations between exposures and outcomes. This method circumvents potential biases and enhances the robustness of causal inferences^37^. According to Mendelian laws of inheritance, parents randomly assign alleles to their offspring. Consequently, MR studies are impervious to the influence of confounding factors^38^. Furthermore, because genes are expressed earlier than the exposure, MR studies avoid reverse causation^39^. We conducted a two-sample MR analysis using aggregated data from expansive genome-wide association studies (GWAS) encompassing schizophrenia, BD, MDD, ADHD, ASD, PD, EC, GC, and CRC. This study aimed to elucidate the causal associations between six psychiatric disorders and the risk of EC, GC, and CRC.

## Methods

### Study design

Two-sample MR investigations were performed using datasets sourced from the publicly accessible GWAS catalogue (https://www.ebi.ac.uk/gwas/) and Psychiatric Genomics Consortium (PGC) (https://pgc.unc.edu/). Ethical approval was deemed unnecessary because it relies solely on preexisting data. This study only investigated European groups, and other populations need to be further investigated in the future.

**Figure 1:**
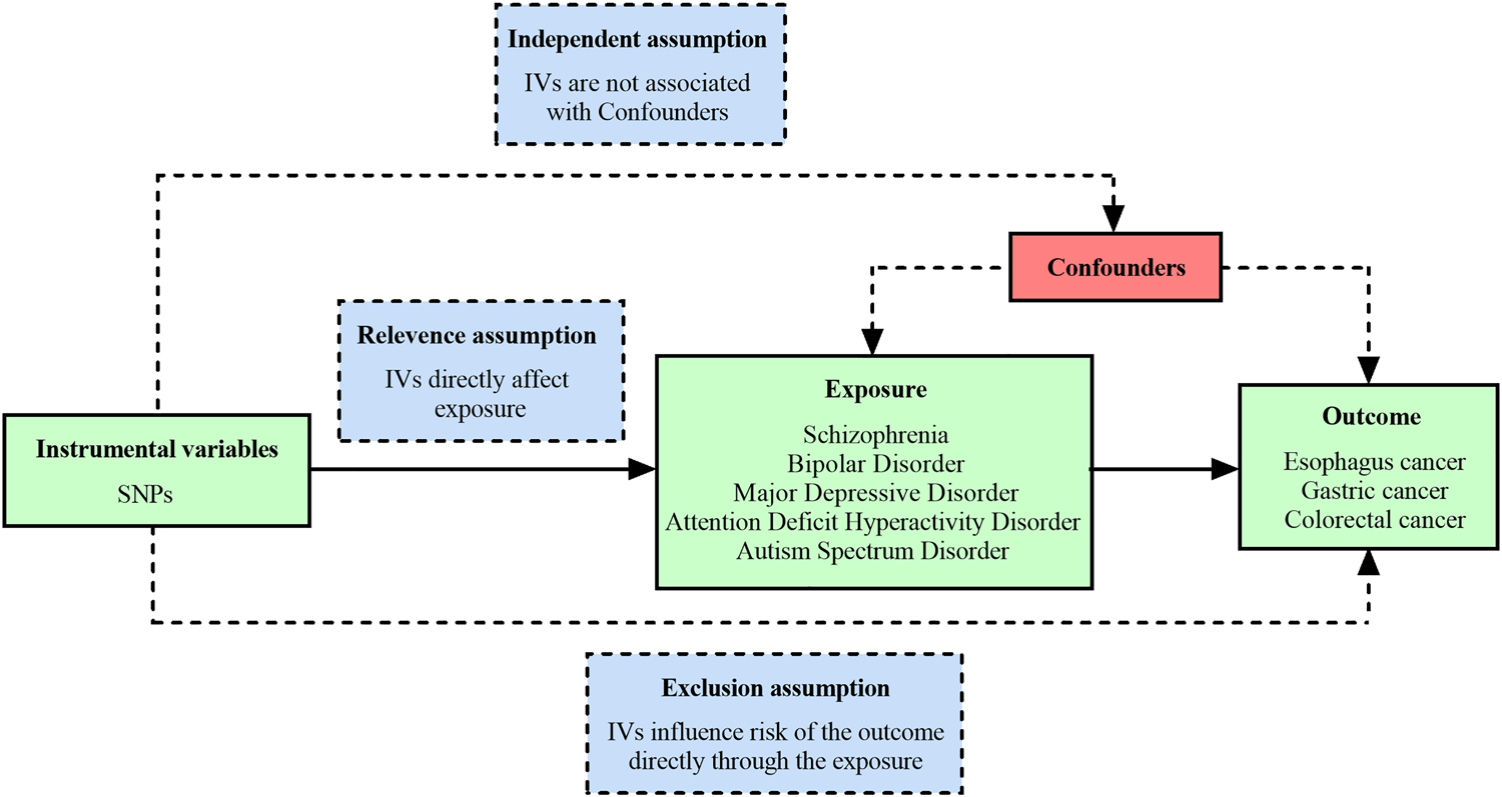
The design of MR analysis was to explore the causal effects between psychiatric disorders and digestive tract cancers. The instrumental variables (IVs) were multiple single-nucleotide polymorphisms (SNPs) linked to the psychiatric disorders, the risk factors were schizophrenia, bipolar disorder, major depressive disorder, attention deficit hyperactivity disorder, autism spectrum disorder, and panic disorder, the outcome variable was esophagus cancer, gastric cancer, and colorectal cancer.

MR analysis integrates the influence of a series of SNPs closely related to exposure factors on outcome events to obtain the causal relationship between exposure factors and outcomes. The MR analysis needs to meet the following three core assumptions^40^: (1) Association hypothesis: the IVs chosen must exhibit a robust correlation with exposure (schizophrenia, BD, MDD, ADHD, ASD, and PD). (2) Exclusivity hypothesis: Genetic IVs should influence outcome factors exclusively through exposure factors. (3) Independence hypothesis: The selected IVs should solely correlate with exposure factors and should not be linked to potential confounding variables. An overview of the study design is shown in Fig. 1.

### Diagnosis of psychiatric disorders and digestive tract cancers

The diagnosis of psychiatric disorders mainly depends on structured clinical interviews and standardised diagnostic criteria, such as those outlined in the DSM-5 (Diagnostic and Statistical Manual of Mental Disorders, Fifth Edition) or ICD-11 (International Classification of Diseases, Eleventh Revision).

Digestive tract cancers can be diagnosed using endoscopy, radiological imaging, histopathological examinations, laboratory tests, and genetic testing.

### GWAS data

Genetic data on psychiatric disorders were obtained from the PGC database. The IVs associated with schizophrenia were obtained from a recent GWAS meta-analysis involving 320,404 participants (76,755 cases, 243,649 controls) of European ancestry^41^. The IVs for BD were collated from a GWAS meta-analysis of 413,466 participants (41,917 cases and 371,549 controls) of European ancestry^42^. The IVs for MDD were acquired through a meta-analysis of the three largest genome-wide depression association studies, incorporating 807,553 European participants (246,363 cases and 561,190 controls)^43^. ADHD IVs were derived from a GWAS meta-analysis involving 225,534 European participants (38,691 cases and 186,843 controls)^44^. ASD IVs data were obtained from a meta-analysis of 14 cohort studies involving 15,954 participants (7,387 cases and 8,567 controls) of European ancestry^45^. Finally, the IVs for PD were pooled from the largest GWAS meta-analysis to date, involving 10,240 European participants (2,248 cases and 7,992 controls)^46^.

The latest GWAS summary data for EC, GC, and CRC were obtained from the GWAS catalogue. The data pertaining to EC included 456,276 individuals of European descent (196 cases and 456,080 controls)^47^. Data for GC included 456,348 European participants (145 cases and 456,203 controls)^47^. CRC data encompassed a collection of 456,276 individuals of European descent (636 cases and 455,640 controls)^47^. The GWAS summary data are presented in Table 1. Although the GWAS data we used were from authoritative databases, there were still relatively few samples, and the results of the study should be reverified in larger samples in the future.

**Table 1:** The GWAS summary data in this Mendelian randomization study.

Year	Trait	Population	Cases	Controls	Samplesize	Websource
2022	Schizophrenia	European	76,755	243,649	320,404	10.1038/s41586-022-04434-5
2021	BD	European	41,917	371,549	413,466	10.1038/s41588-021-00857-4
2019	MDD	European	246,363	561,190	807,553	10.1038/s41593-018-0326-7
2023	ADHD	European	38,691	186,843	225,534	10.1038/s41588-022-01285-8
2017	ASD	European	7387	8567	15,954	10.1186/s13229-017-0137-9
2019	PD	European	2248	7992	10,240	10.1038/s41380-019-0590-2
2021	EC	European	196	456,080	456,276	10.1038/s41588-021-00954-4
2021	GC	European	145	456,203	456,348	10.1038/s41588-021-00954-4
2021	CRC	European	636	455,640	456,276	10.1038/s41588-021-00954-4

*GWAS* genome-wide association studies, *BD* bipolar disorder, *MDD* major depressive disorder, *ADHD* attention deficit hyperactivity disorder, *ASD* autism spectrum disorder, *PD* panic disorder, *EC* esophagus cancer, *GC* gastric cancer, *CRC* colorectal cancer.

### Selection of IVs

The SNPs that met the three hypotheses were selected based on the following criteria: (1) Association hypothesis: All IVs must attain gene-wide significance, denoted by P < 5 × 10^−8^. However, for ASD and PD, the threshold was too high to include IVs. Therefore, for these specific conditions, the threshold was relaxed to *P* < 5 × 10^−6^. This inevitably led to a reduction in the credibility of the results. The F-value, representing the strength of the MR analysis, is a crucial indicator. F > 10 signifies the robust predictive power of the IVs for exposures. The F-value is calculated as follows: F = R^2^ (n – k − 1)/[k (1 − R^2^)], R^2^ = 2 × EAF × (1 − EAF) × β^2^^48^. (2) Exclusivity hypothesis: The linkage disequilibrium parameter (R^2^) should be less than 0.001, and the genomic region span should be confined within 10,000 kilobases. (3) Independence hypothesis: search the IVs individually in the PhenoScanner database to exclude SNPs that show strong correlations with other exposure factors^49^. These criteria ensured the precision and reliability of the IVs used in this study.

### Statistical analysis

The analytical approach employed in this study primarily embraces the (inverse variance weighted) IVW method, leveraging meta-analytical techniques for the amalgamation of the Wald ratios attributed to individual SNPs. Implicit in this method is the assumption that IVs exert an impact on outcomes exclusively through designated exposures. Consequently, the IVW method yielded robust results in the absence of polymorphism^50^. The IVW method utilises the inverse of the variance associated with each IV for weight computation. This procedure was done to ensure the validity of all IVs, thereby facilitating the evaluation of horizontal pleiotropy^51^. Nevertheless, uncertainties in genetic associations and risk factors, such as the presence of weak IVs, introduce bias into the IVW method, resulting in an underestimation of the actual results^52^. To address these potential limitations, supplementary analyses were conducted using the MR-Egger regression and weighted median (WM) methods. The MR-Egger regression incorporates the inverse of the outcome variance as a weighting factor to fit the model. Simultaneously, it introduces intercept terms during the regression, enabling weighted linear regression in instances in which genetic IVs are invalid, thereby generating causal estimates^53^. The WM method, defined as the median of the weighted empirical density function of the ratio, estimates the amalgamated data from multiple genetic variants to derive a singular causal estimate. The WM method consistently provides effect estimates even when the proportion of invalid genetic IVs is as high as 50% and when there is substantial variability in the accuracy of estimates among IVs^54^.

To assess the robustness and reliability of our study rigorously, we conducted a comprehensive examination involving quality control, sensitivity analysis, heterogeneity testing, and gene-level pleiotropy testing. A leave-one-out analysis was used to assess the sensitivity of the study results. This method systematically excludes each IV (SNP) and subsequently computes results based on the remaining IVs. The absence of statistically significant differences between the outcomes of individual IVs and the overall results indicated the absence of a nonspecific effect on the effect estimation results^55^. Cochran's Q test was used to quantify the heterogeneity of IVs. *P* > 0.05 indicated no significant heterogeneity was observed, leading to the predominant use of the fixed-effects IVW method. Conversely, *P* < 0.05 signified apparent heterogeneity, leading to the adoption of the random-effect IVW method^56^. Funnel plots served as tools to discern the presence of publication bias. The approximate symmetry of the plots suggests no obvious publication bias. Detection of horizontal pleiotropy was facilitated by MR-Egger regression. Horizontal pleiotropy was deemed absent when the intercept terms exhibited minimal deviation from 0, and the associated *P* > was 0.05^53^. The MR-pleiotropy Residual Sum and Outlier (MR-PRESSO) method was used to remove significant outliers and reduce horizontal pleiotropy^57^.

### Ethical approval

Our research is an analysis of previous data and does not involve human participants or animals. No additional ethical approval was required due to the re-analysis of previously summary-level data. This study was conducted according to the guidelines of the Declaration of Helsinki. The database, used for analysis, contains anonymized electronic patient data. Patient data was analyzed in aggregated form without the inclusion of individual data. An informed consent form was not obtained in adherence with national and European legislation.

## Results

### Genetic IVs for Schizophrenia, BD, MDD, ADHD, ASD, and PD

For schizophrenia, 26 significant (*P* < 5 × 10^–8^) and independent (R^2^ < 0.001) SNPs were included as genetic IVs. The IVs demonstrated a robust predictive capacity for outcomes, as evidenced by a minimum F-value surpassing 30, which well exceeded the threshold of 10. In the context of BD, 52 SNPs were employed as IVs, each exhibiting an F-value > 31. Similarly, MDD featured 53 SNPs as IVs, with each F-value of > 30. ADHD involved 26 SNPs as IVs, with each F-value > 30. ASD incorporated 10 SNPs as IVs, characterized by F-values > 21. Finally, PD encompassed 13 SNPs as IVs, each with each F-value > 21. The details of the SNPs associated with schizophrenia, BD, MDD, ADHD, ASD, and PD are shown in Tables S1.1–S1.6. The SNPs at the intersection of psychiatric disorders and digestive tract cancers are presented in Tables S2.1–S2.18.

### Causal effect from psychiatric disorders to EC risk

**Figure 2:**
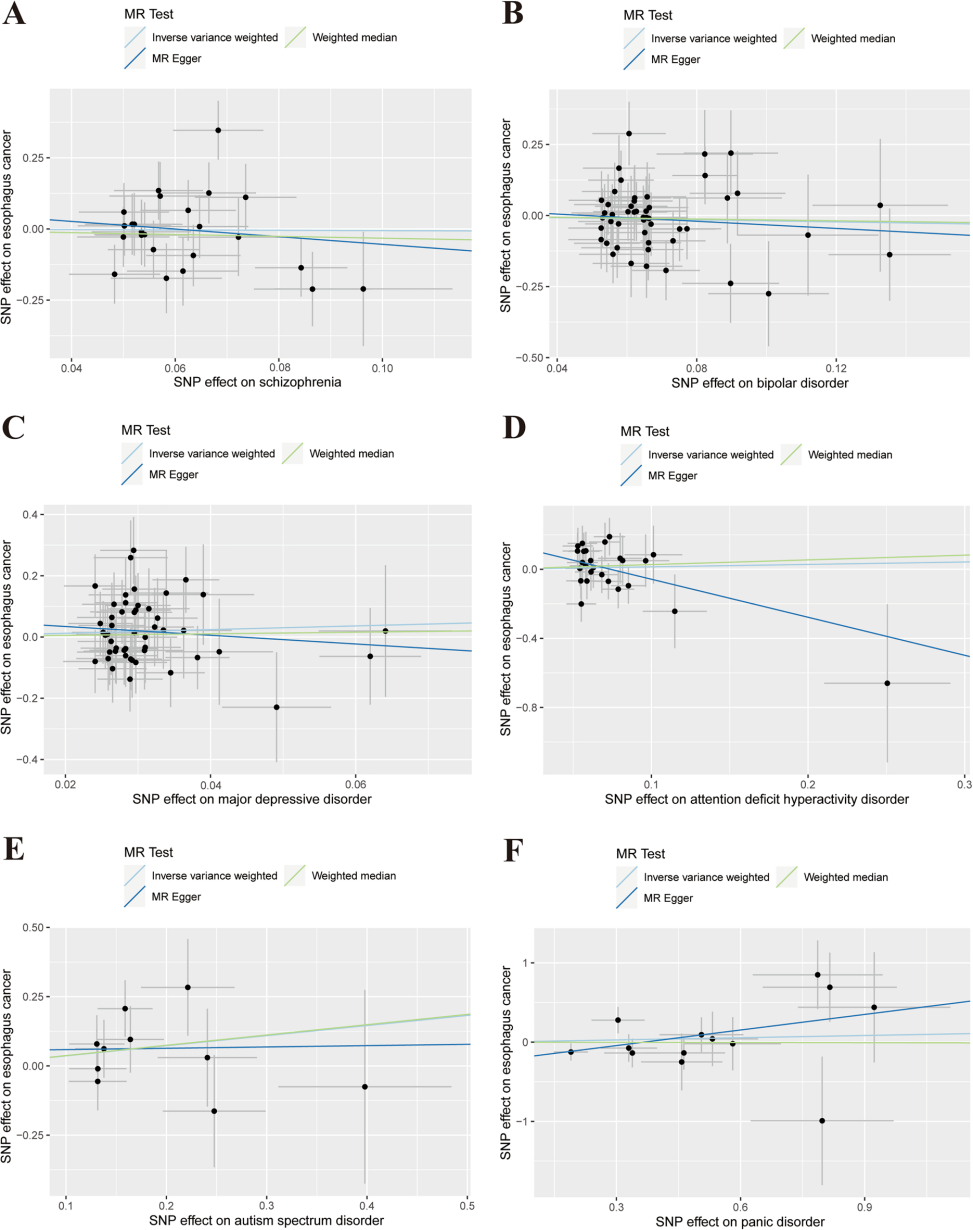
Scatter plot depicting psychiatric disorders and esophagus cancer. (**A**) Schizophrenia, (**B**) bipolar disorder, (**C**) major depressive disorder, (**D**) attention deficit hyperactivity disorder, (**E**) autism spectrum disorder, (**F**) panic disorder. Each of these points represents an instrumental variable. The vertical and horizontal lines at the center of the dot represent 95% CI. The slope of the colored line represents the size of the causal relationship.

The results of the MR analysis are presented in Table 2. The IVW test showed that there was no causal relationship between schizophrenia [odds ratio (OR) = 0.94, 95% confidence interval (CI) 0.41–2.15, *P* = 0.89], BD (OR = 0.83, 95% CI 0.51–1.34, *P* = 0.45), MDD (OR = 1.83, 95% CI 0.64–5.23, *P* = 0.26), ADHD (OR = 1.15, 95% CI 0.60–2.21, *P* = 0.67), ASD (OR = 1.44, 95% CI 0.90–2.31, *P* = 0.13), PD (OR = 1.10, 95% CI 0.79–1.533, *P* = 0.58), and EC risk. The results of the WM and MR-Egger methods were consistent with those of the IVW method. Given the absence of notable heterogeneity and apparent horizontal pleiotropy, we deemed the results of the IVW test more credible. The results remained consistent after multiple corrections using the FDR and Bonferroni methods. Table 3 shows the adjusted *P*-values after multiple corrections using the FDR and Bonferroni methods. Scatter plots depict the causal estimates derived from each instrumental variable (Fig. 2). Cochran’s Q test showed that the P-values of all results were greater than 0.05, indicating no obvious heterogeneities (Table S3). The MR-Egger regression showed that the intercept term of each result was very close to 0, and all the P-values were greater than 0.05. Therefore, no horizontal pleiotropy was observed in any of the MR analyses (Table S4). No significant outliers required elimination using the MR-PRESSO method. The funnel plot for each MR analysis was relatively symmetrical, indicating no obvious bias (Fig. S1). The leave-one-out analysis confirmed the stability of MR estimation after eliminating a single SNP (Fig. S2).

**Table 2:**
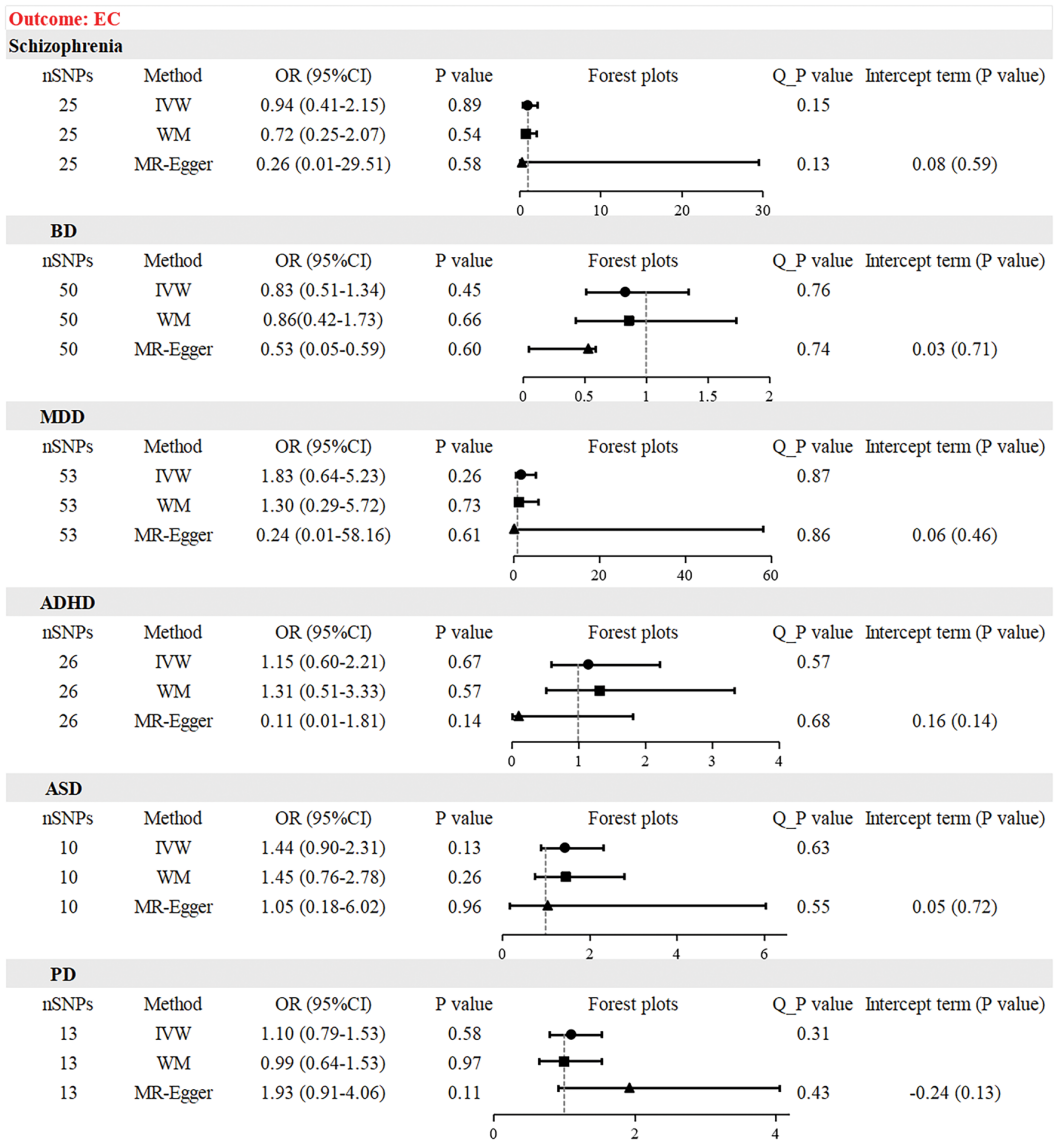

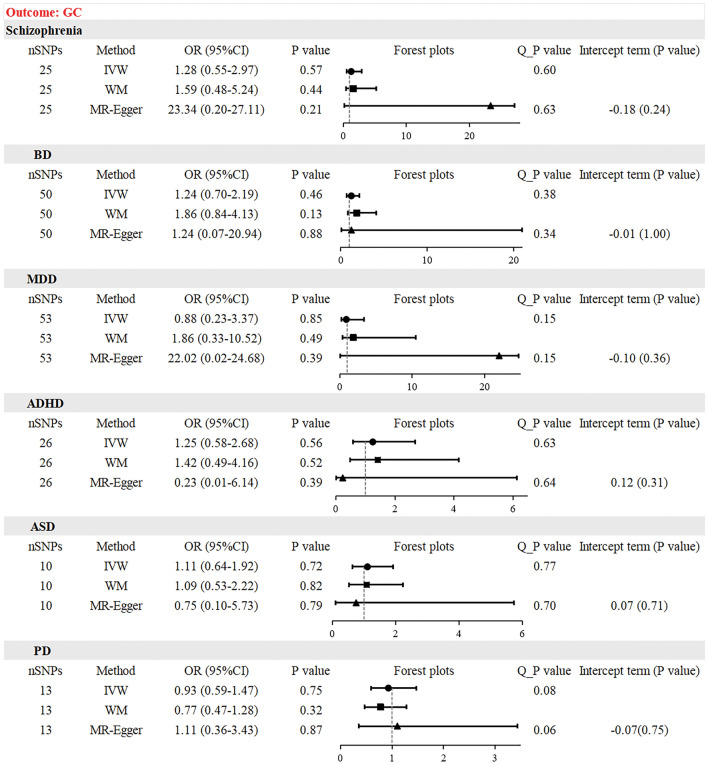

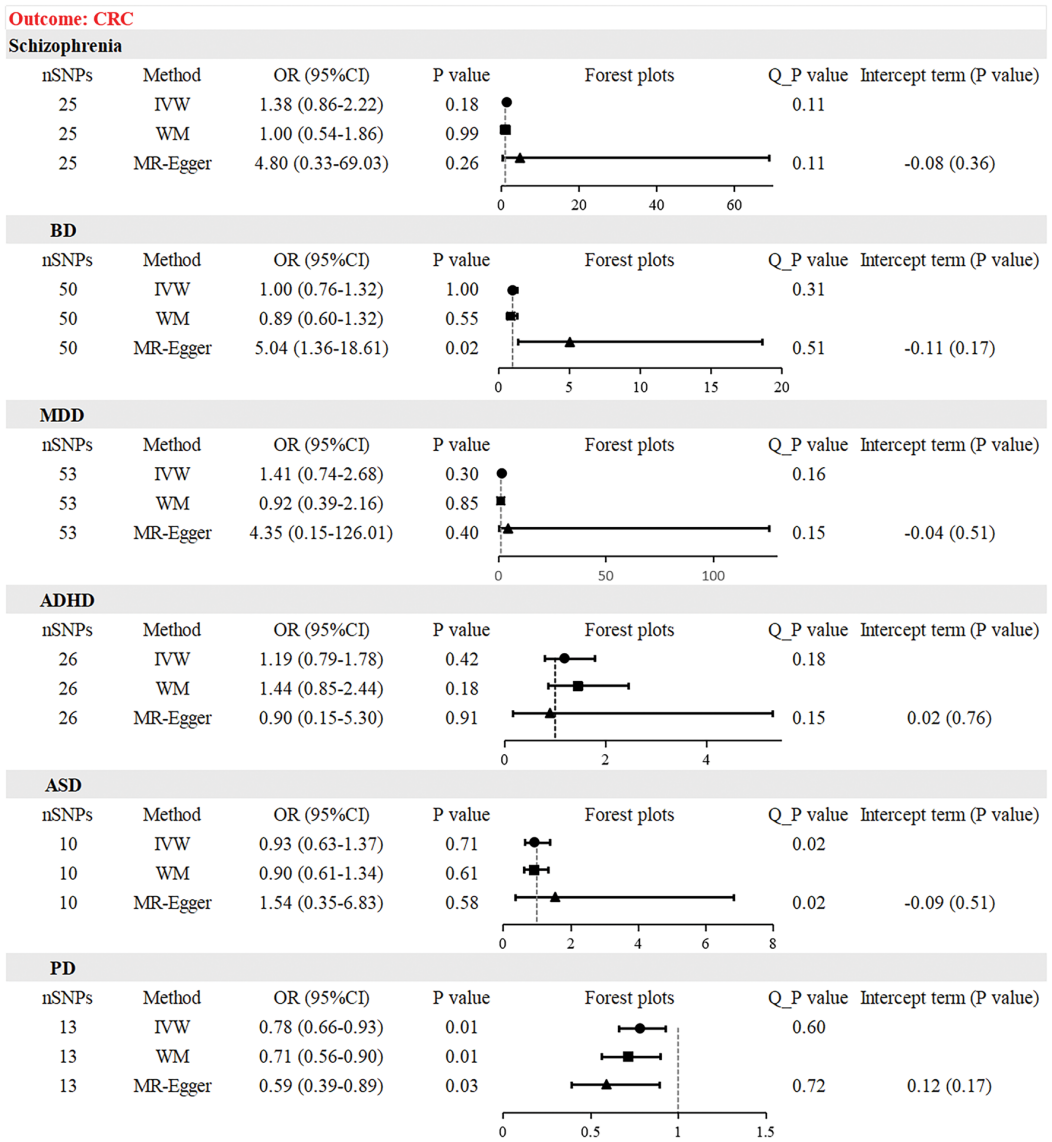
Causal associations between six psychiatric disorders and the risks of digestive tract cancers.

*BD* bipolar disorder, *MDD* major depressive disorder, *ADHD* attention deficit hyperactivity disorder, *ASD* autism spectrum disorder, *PD* panic disorder, *EC* esophagus cancer, *GC* gastric cancer, *CRC* colorectal cancer.

**Table 3:** The adjusted P values after the multiple corrections using the FDR and Bonferroni methods.

	EC		GC		CRC	
	FDR method	Bonferroni method	FDR method	Bonferroni method	FDR method	Bonferroni method
Schizophrenia	0.89	1.00	0.85	1.00	0.54	1.00
BD	0.81	1.00	0.85	1.00	1.00	1.00
MDD	0.79	1.00	0.85	1.00	0.59	1.00
ADHD	0.81	1.00	0.85	1.00	0.62	1.00
ASD	0.79	0.79	0.85	1.00	0.85	1.00
PD	0.81	1.00	0.85	1.00	0.03	0.03

The adjusted P values were obtained based on the P values from IVW method.

*GWAS* genome-wide association studies, *BD* bipolar disorder, *MDD* major depressive disorder, *ADHD* attention deficit hyperactivity disorder, *ASD* autism spectrum disorder, *PD* panic disorder, *EC* esophagus cancer, *GC* gastric cancer, *CRC* colorectal cancer.

### Causal effect from psychiatric disorders to GC risk

**Figure 3:**
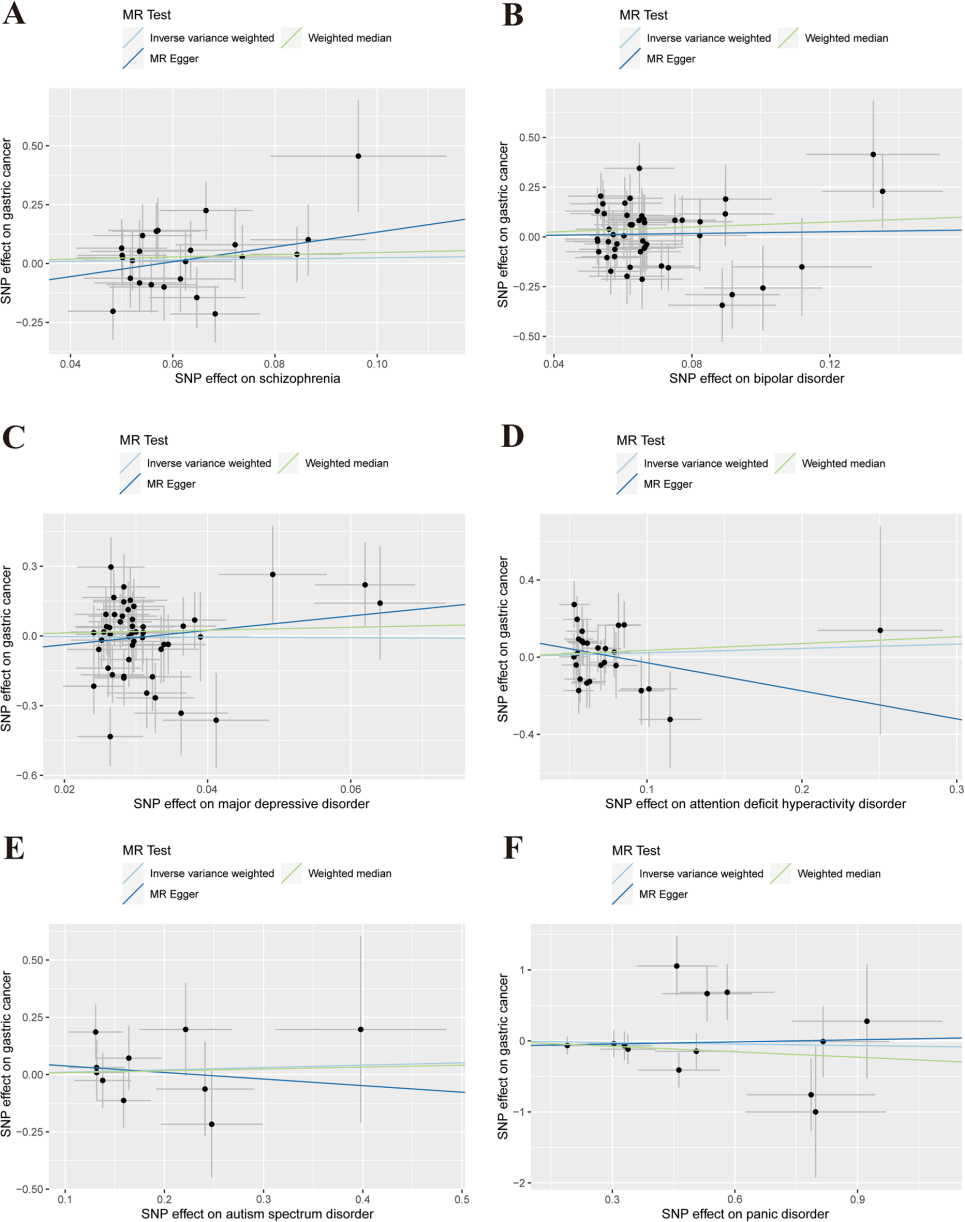
Scatter plot depicting psychiatric disorders and gastric cancer. (**A**) schizophrenia, (**B**) bipolar disorder, (**C**) major depressive disorder, (**D**) attention deficit hyperactivity disorder, (**E**) autism spectrum disorder, (**F**) panic disorder. Each of these points represents an instrumental variable. The vertical and horizontal lines at the center of the dot represent 95% CI. The slope of the colored line represents the size of the causal relationship.

Table 2 presents the results of the MR analysis. The IVW test asserted the absence of evidence for a causal relationship between schizophrenia (OR = 1.28, 95% CI 0.55–2.97, *P* = 0.57), BD (OR = 1.24, 95% CI 0.70–2.19, *P* = 0.46), MDD (OR = 0.88, 95% CI 0.23–3.37, *P* = 0.85), ADHD (OR = 1.25, 95% CI 0.58–2.68, *P* = 0.56), ASD (OR = 1.11, 95% CI 0.64–1.92, *P* = 0.72), PD (OR = 0.93, 95% CI 0.59–1.47, *P* = 0.75), and the risk of GC. Consistent findings were observed in the WM and MR-Egger tests. The Scatter plots are shown in Fig. 3. The results of multiple corrections using the FDR and Bonferroni methods were consistent with previous results (Table 3). The P-values of Cochran's Q test were all greater than 0.05 (Table S5). No obvious horizontal pleiotropies were detected in the MR-Egger regression analysis (Table S6). A symmetrical funnel plot showed no obvious biases did not exist (Fig. S3). The leave-one-out analysis showed that a single IV did not have a nonspecific effect on the effect estimation, and the results of each MR analysis were stable (Fig. S4).

### Causal effect from psychiatric disorders to CRC risk

**Figure 4:**
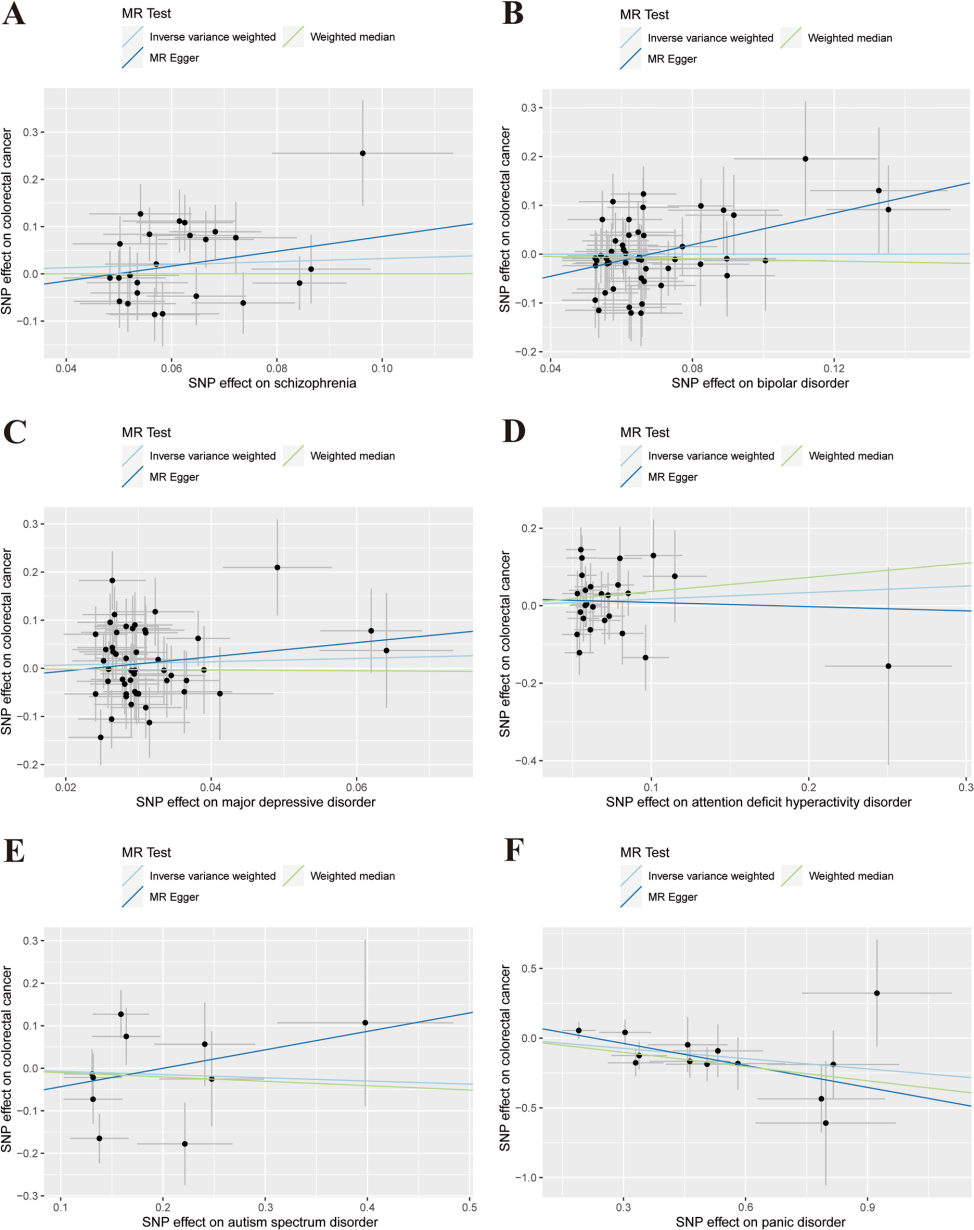
Scatter plot depicting psychiatric disorders and colorectal cancer. (**A**) schizophrenia, (**B**) bipolar disorder, (**C**) major depressive disorder, (**D**) attention deficit hyperactivity disorder, (**E**) autism spectrum disorder, (**F**) panic disorder. Each of these points represents an instrumental variable. The vertical and horizontal lines at the center of the dot represent 95% CI. The slope of the colored line represents the size of the causal relationship.

The test of IVW results showed that schizophrenia (OR = 1.38, 95% CI 0.86–2.22, *P* = 0.18), BD (OR = 1.00, 95% CI 0.76–1.32, *P* = 1.00), MDD (OR = 1.41, 95% CI 0.74–2.68, *P* = 0.30), ADHD (OR = 1.19, 95% CI 0.79–1.78, *P* = 0.42) and ASD (OR = 0.93, 95% CI 0.63–1.37, *P* = 0.71) were not genetic risk factors for CRC. The WM and MR-Egger methods yielded similar results (Table 2). However, for BD, the MR-Egger test showed a different result (OR = 5.04, 95% CI 1.36–18.61, *P* = 0.02). This suggests a weak positive correlation between BD and CRC risk, but the IVW results were more credible. In terms of PD, The MR analysis suggested a negative association between PD and CRC risk, as indicated by IVW test (OR = 0.78, 95% CI 0.66–0.93, *P* = 0.01), with a consensus corroborated by the WM test (OR = 0.71, 95% CI 0.56–0.90, *P* = 0.01) and the MR-Egger test (OR = 0.59, 95% CI 0.39–0.89, *P* = 0.03). PD has been postulated to be a protective factor against CRC. The scatter plot showed a negative association (Fig. 4F). After multiple corrections using FDR and Bonferroni methods, all results remained consistent (Table 3). Cochran’s Q test did not detect significant heterogeneity in the results (Table S7). The MR-Egger regression showed that no obvious horizontal multiplicity existed in each MR analysis (Table S8). The funnel plots were relatively symmetrical (Fig. S5). The leave-one-out analysis confirmed the stability of each MR analysis (Fig. S6).

## Discussion

The results indicated that psychiatric disorders exhibited little association with genetic risk factors for digestive tract cancers. Notably, a negative association was identified between PD and CRC risk.

Regarding the association between schizophrenia and cancers, it is well established that individuals with schizophrenia exhibit a low incidence of cancer and demonstrate potential immunity. This intriguing phenomenon has garnered significant attention from researchers, leading to numerous large-scale population-based studies consistently indicating markedly lower cancer rates in individuals with schizophrenia than in the general population^58,59^. Some of these investigations propose the presence of specific "protective factors" in people with schizophrenia, shielding them from cancer. However, a recent study has shown that people with schizophrenia have a higher incidence of tumours^60^. Meanwhile, a MR analysis has refuted any causal link between schizophrenia and prostate cancer risk^61^. Our study provided new insights into these contradictory results. The results do not support the notion that schizophrenia increases the risk of cancer. Recent research has revealed that antipsychotic drugs used in schizophrenia treatment may exert inhibitory effects on cancers^62^. For instance, the psychotropic drugs of the diphenylbutylpiperidine class, including fluspirilene, penfluridol, and pimozid, exert inhibitory effects on tumour suppressor protein phosphatase 2A down-regulation, AKT, GSK3b, MYC, p70 S6K ubiquitination, and degradation. This multifaceted molecular modulation culminates in tumour cell death^63^. Moreover, these compounds can induce M1 polarisation in macrophages, thereby modulating the tumour immune microenvironment^64^. Concurrently, anti-schizophrenia medications contribute to impeding tumour cell invasion and metastasis, fostering tumour cell apoptosis, and inhibiting tumour cell autophagy^65^. In addition, some studies showed that patients with schizophrenia are more likely to develop tumours, which can be attributed to fewer opportunities for physical care and their physical diseases^33^.

Regarding BD, a meta-analysis of nine observational studies indicated an elevated risk of cancer associated with BD^18^. However, our study presents a different perspective, revealing no substantial evidence of a causal relationship between BD and GC risk. The inherent limitations of observational studies, marked by the mitigation of confounding variables, frequently yield inconclusive outcomes. Plausible factors contributing to the observed positive correlation include physicians' reluctance to advocate cancer screening for patients with BD, attribution of early cancer symptoms to BD by medical professionals, patients’ non-compliance with cancer screening protocols, cognitive deficits leading to delayed access to healthcare information, and the influence of the patients’ unhealthy lifestyles, such as smoking, alcohol consumption, unbalanced diet, and low consumption of fruits and vegetables^66^.

Prior investigations have yielded inconclusive findings regarding the association between MDD and cancer risk, with divergent perspectives on the causal relationship. While certain studies suggest an increased risk of cancer in MDD^22^, others contest the existence of a direct link^24^. However, the outcomes of our study indicate that MDD is not a risk factor for EC, GC, and CRC. Despite our findings suggesting that MDD is not directly linked to tumour risk, alternative pathways may still be involved in cancer progression. Notably, MDD's impact on the endocrine system has been highlighted, with disruptions in hormone levels potentially elevating susceptibility to various cancers, including breast and digestive tract cancers^67^. Moreover, MDD may indirectly increase the risk of cancer by impeding the proliferation of natural killer cells and inducing the inactivation of DNA repair enzymes^68^. Emerging research suggests that MDD dysregulates inflammatory cytokines such as IL-6, thereby potentially fostering cancers^69^.

Few studies have explored the relationship between ADHD and cancer. A recent retrospective cohort study encompassing 798 cases reported an elevated risk of CRC associated with ADHD^27^. However, our study yielded contradictory results. Given the substantial information bias inherent in retrospective studies, the association between ADHD and CRC warrants validation through additional prospective and randomized controlled studies. In the absence of such investigations, MR analysis is a more dependable approach, being less susceptible to bias. These observations can be attributed to various factors. First, the unhealthy lifestyle of individuals with ADHD, characterised by smoking, alcohol consumption, and obesity, may serve as potential risk factors for CRC^70–72^. Second, the socioeconomic disadvantages experienced by individuals with ADHD may indirectly contribute to the incidence of CRC^73^.Lastly, Individuals with ADHD exhibit an increased propensity, compared to the general population, to experience inflammatory conditions, such as asthma, eczema, and ankylosing spondylitis^74–76^.Notably, inflammation has been substantiated as a notable risk factor for the development of CRC^77^.

Divergent perspectives exist regarding ASD and cancer. Some scholars have posited that there is no causal relationship between ASD and cancers, which is consistent with our results^29^. Others have contended that an association between ASD and cancers may exist^30^. A recent investigation revealed that individuals with ASD who are diagnosed with cancer frequently present with intellectual disabilities or birth defects^78,79^. Notably, this subgroup demonstrated a heightened cancer risk compared to individuals with only ASD. As the genes considered in the MR study exclusively influenced the outcomes through exposure factors and remained unaffected by other confounding variables, we posit that the findings of this study are inherently more precise.

The association between PD and cancer has been hardly researched. Therefore, we present an initial attempt to elucidate the association between PD and digestive tract cancers. These findings imply an inverse association between PD and CRC risk, indicating that PD may mitigate the risk of CRC. However, the mechanism by which PD affects CRC risk remains unexplored. We speculate that genes in individuals with PD may play a role in intricate physiological pathways, including immune activation or apoptosis. Nevertheless, a thorough investigation is required to validate this.

Compared with a previous MR study on the relationship between mental illness and breast cancer, we found that some of the confidence intervals for the ORs in MR-egger analysis were wide in our study^32^. This may be related to the following reasons: First, the sample size of patients with cancer in this study was smaller than that in the previous study, which affected the confidence intervals. In addition, patients with cancer may also affect the confidence intervals; patients with breast cancer were mainly middle-aged women, whereas patients with digestive tract tumours were men and women of all ages. Furthermore, compared with other methods, MR-egger test was more conservative and had lower statistical efficiency.

Although our study indicates that most psychiatric disorders are not associated with digestive tract cancers, we offer genetic insights to explain previous findings. The inconsistencies may be attributed to factors, such as medication, lifestyle, and early cancer screening of patients. These results provide a new basis for understanding the relationship between psychiatric disorders and digestive tract cancers. At the same time, the negative association between PD and CRC can provide new ideas for the follow-up exploration of the pathogenesis and clinical prevention of CRC.

In summary, our study has several advantages. MR analysis stands out for its unique ability to avoid confounding and reverse causality. This makes it particularly valuable for causal inference in genetics, where observational studies often face challenges. Additionally, the accessibility of data sources for MR analysis facilitates a more efficient exploration of causal relationships between diseases. Furthermore, we rigorously screened SNPs to ascertain the significance, independence, and predictive potency of IVs. This meticulous approach enhances the reliability of our MR analysis.

Nevertheless, it is imperative to acknowledge the inherent limitations of this study. First, despite efforts to leverage the most expansive, current, authoritative, and comprehensive GWAS databases available, the sample size remained modest. Second, although we strictly followed the three hypotheses, some behaviours that violated them still existed. For example, because few SNPs could be used in the ASD and PD groups, we appropriately relaxed the threshold of P-value, which violated the association hypothesis. In addition, we could not completely remove confounding factors that violated the independence hypothesis. Therefore, a thoughtful assessment of the degree to which these assumptions could have been violated is ignored, such as the power of the statistical tests used to assess the violations of assumptions. Third, it is imperative to note that the GWAS data utilised in this study exclusively emanated from European populations, necessitating validation of the results across diverse ethnic groups. Fourth, epigenetic challenges encompassing issues, such as RNA editing and DNA methylation, constitute inherent limitations in MR research.

## Conclusion

This study introduces a pioneering MR analysis investigating the causality between psychiatric disorders and digestive tract cancers risk. Our findings suggest that schizophrenia, BD, MDD, ADHD, ASD, and PD do not increase the risk of EC, GC, and CRC. Interestingly, we observed a negative correlation between PD and CRC risk. This genetic evidence contributes to our understanding of the association between psychiatric disorders and susceptibility to gastrointestinal cancer. This study provides genetic evidence elucidating the association between psychiatric disorders and the risk of digestive tract cancers.

### Supplementary Information

Supplementary Figure 1.

Supplementary Figure 2.

Supplementary Figure 3.

Supplementary Figure 4.

Supplementary Figure 5.

Supplementary Figure 6.

Supplementary Table 1.

Supplementary Table 2.

Supplementary Table 3.

Supplementary Table 4.

Supplementary Table 5.

Supplementary Table 6.

Supplementary Table 7.

Supplementary Table 8.

Supplementary Legends.

## Data Availability

The summary statistics of digestive tract cancers were obtained from a GWAS study published by Jiang, L et al. The data associated with schizophrenia was obtained from a GWAS meta-analysis published by Trubetskoy, V et al. The data for BD was collated from a GWAS meta-analysis published by Mullins, N et al. The data for MDD was acquired through a GWAS meta-analysis published by Howard, D et al. The data of ADHD was derived from a GWAS meta-analysis published by Demontis, D et al. The ASD data was obtained from a GWAS meta-analysis published by Anney, R. J. L et al. The data for PD was pooled from a GWAS meta-analysis published by Forstner, A. J et al. The summary data of digestive tract cancers can be obtained on GWAS catalogue (https://www.ebi.ac.uk/gwas/), and the summary data of psychiatric disorders can be collated on Psychiatric Genomics Consortium (https://pgc.unc.edu/).
