# Supplementary figures and images for: Causality between six psychiatric disorders and digestive tract cancers risk: a two-sample Mendelian randomization study
Source: Sci Rep. 2024 Jul 19;14:16689. doi: 10.1038/s41598-024-66535-7 (PMC11271641; doi:10.1038/s41598-024-66535-7)

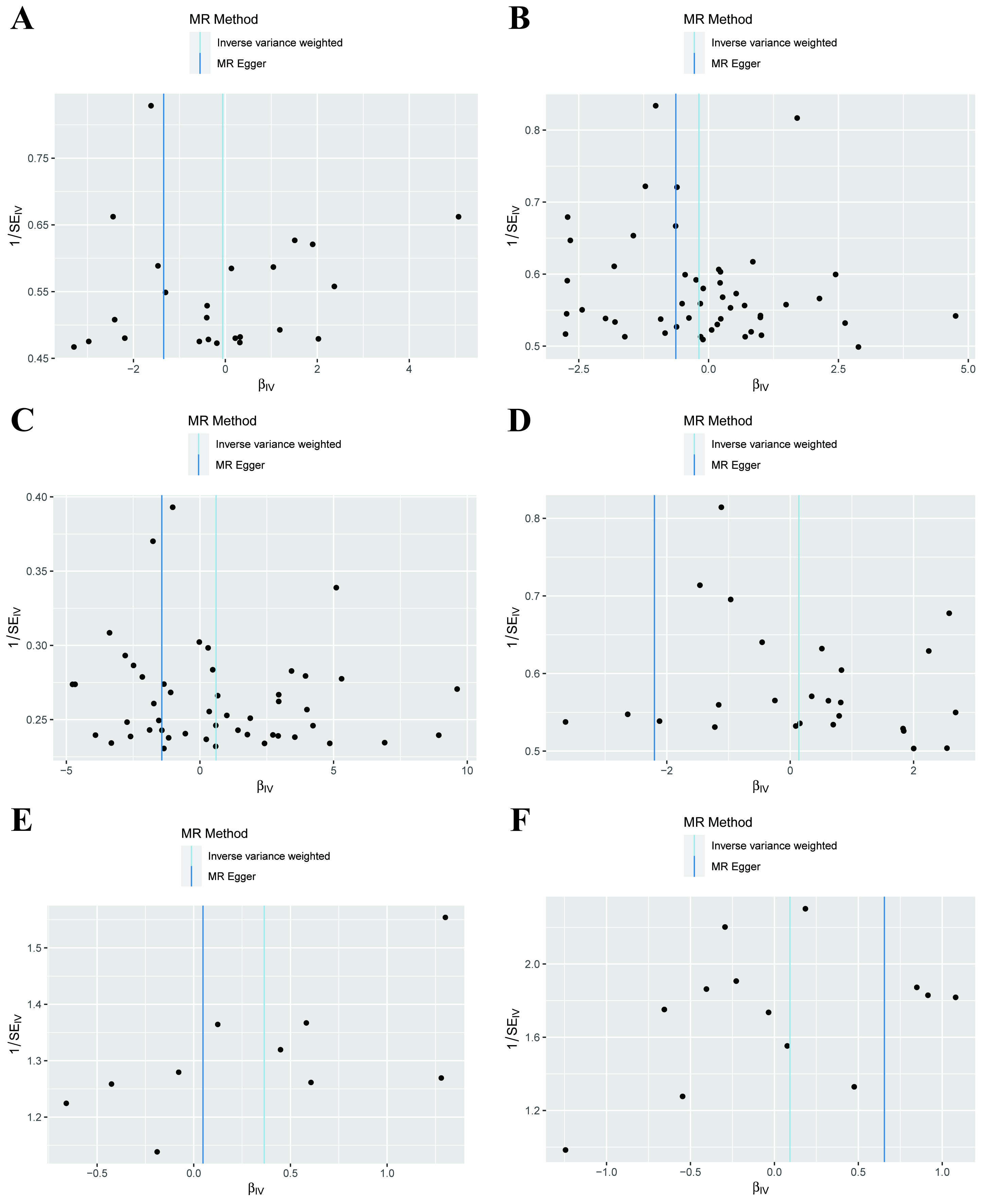

Supplement: Supplementary file 1 — Supplementary Figure 1. [file 41598_2024_66535_MOESM1_ESM.tif]

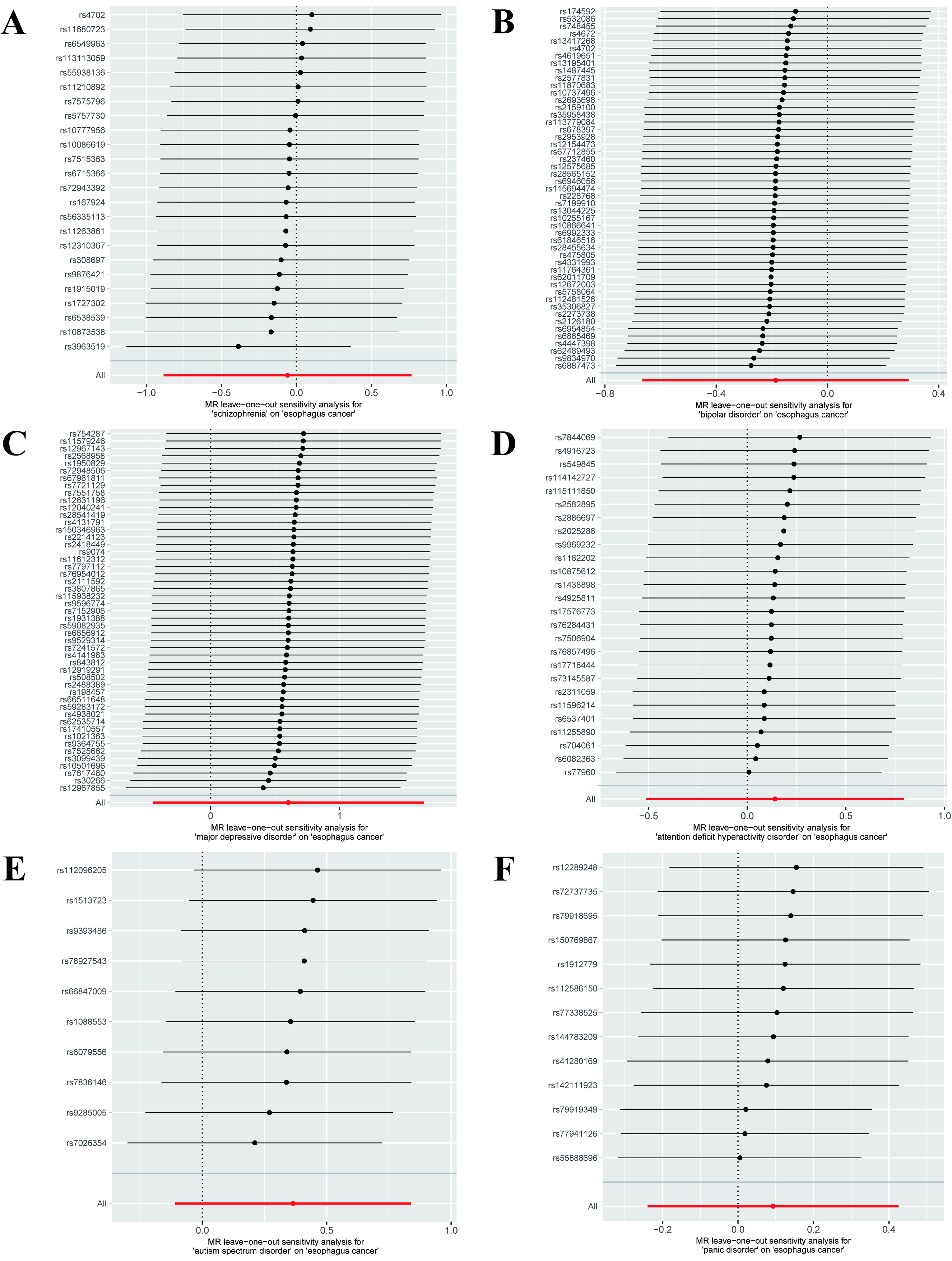

Supplement: Supplementary file 2 — Supplementary Figure 2. [file 41598_2024_66535_MOESM2_ESM.tif]

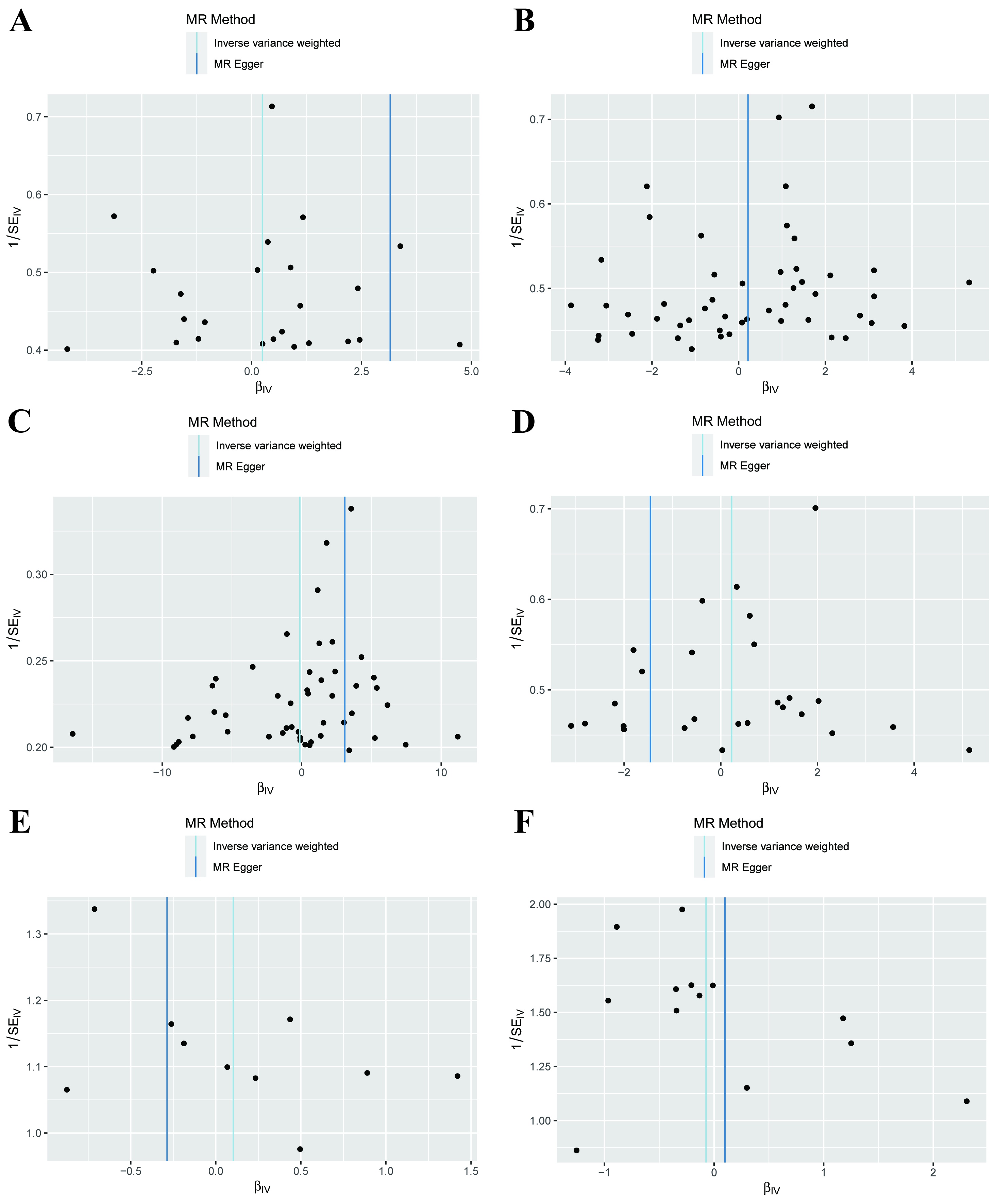

Supplement: Supplementary file 3 — Supplementary Figure 3. [file 41598_2024_66535_MOESM3_ESM.tif]

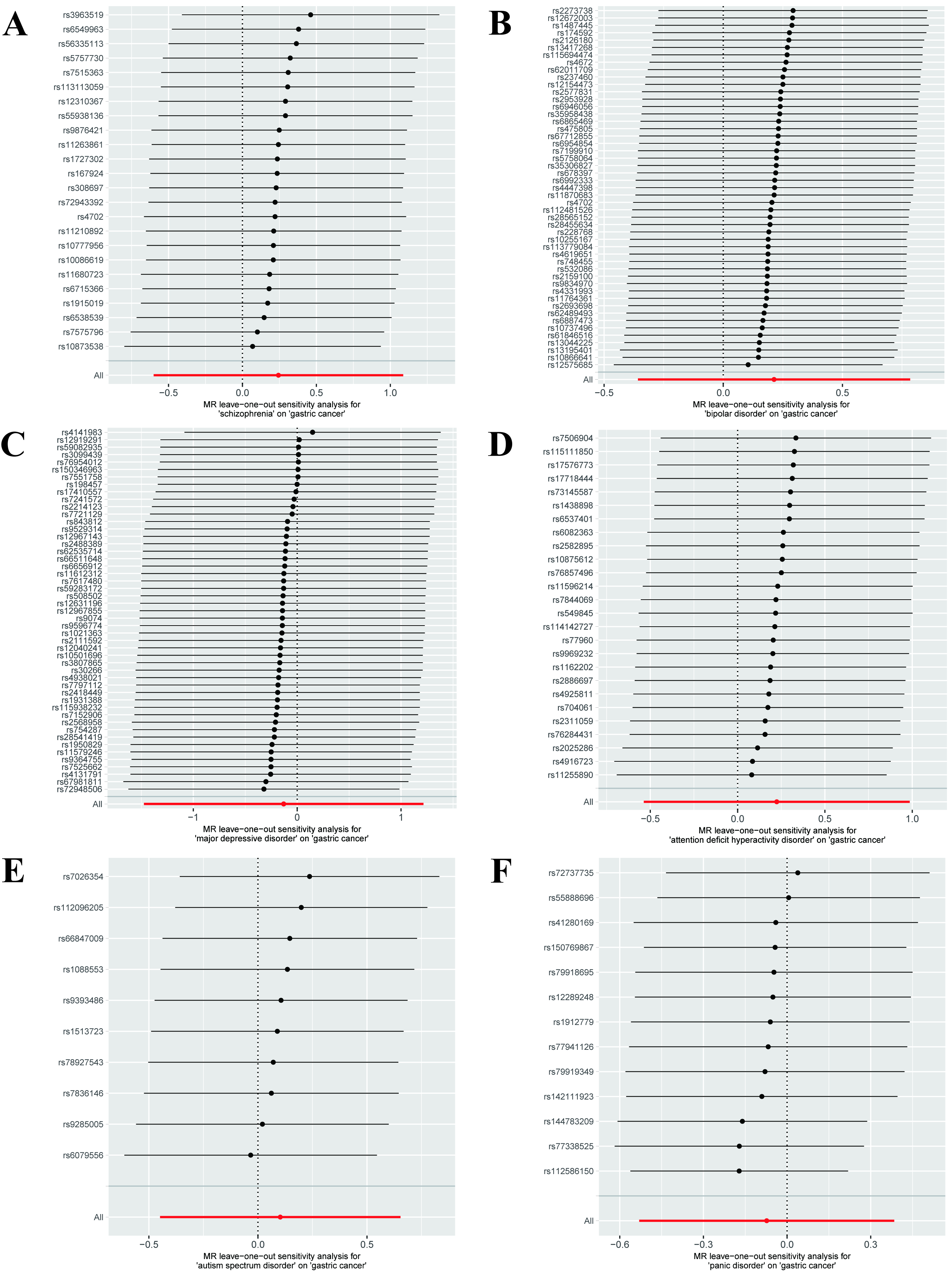

Supplement: Supplementary file 4 — Supplementary Figure 4. [file 41598_2024_66535_MOESM4_ESM.tif]

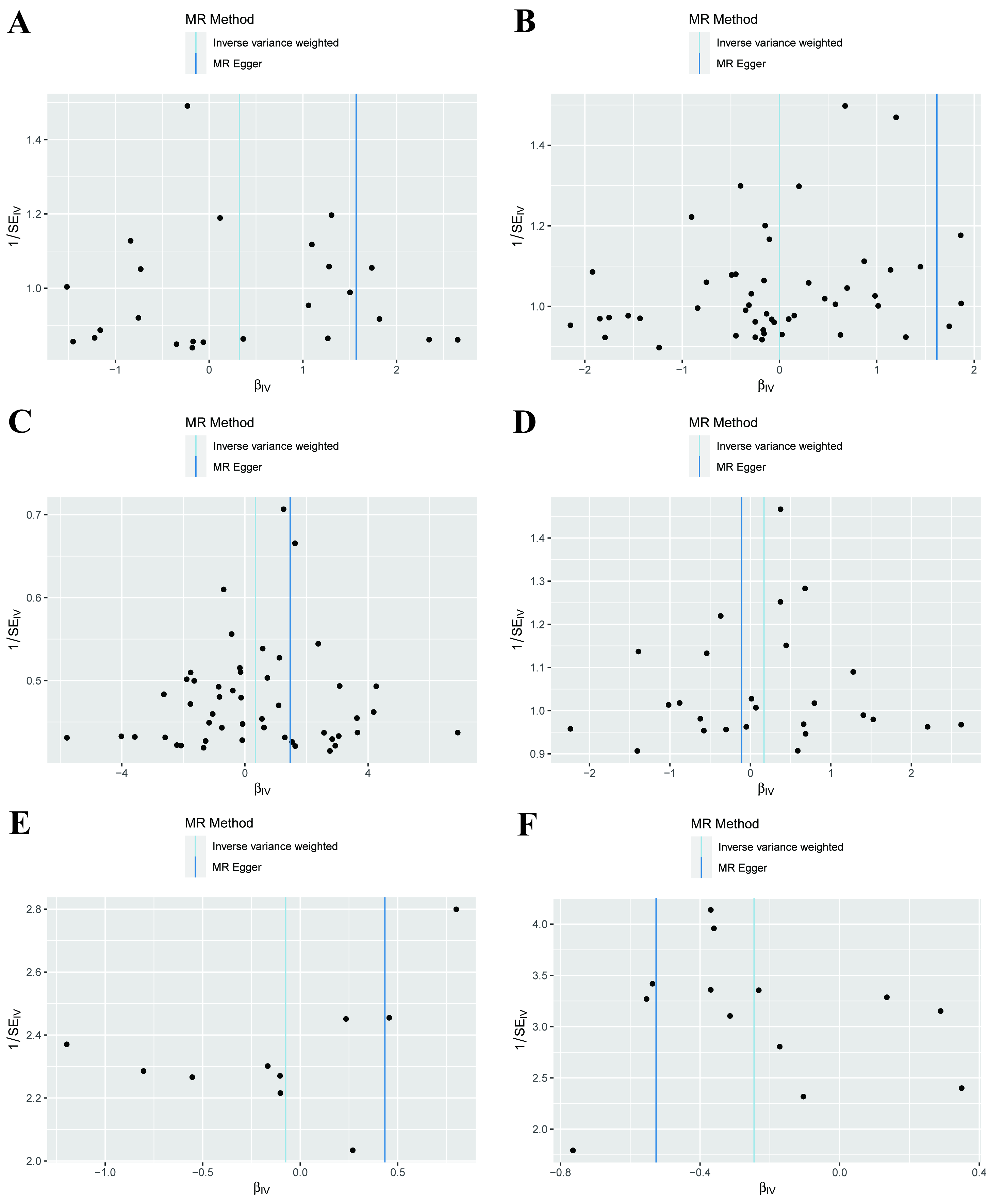

Supplement: Supplementary file 5 — Supplementary Figure 5. [file 41598_2024_66535_MOESM5_ESM.tif]

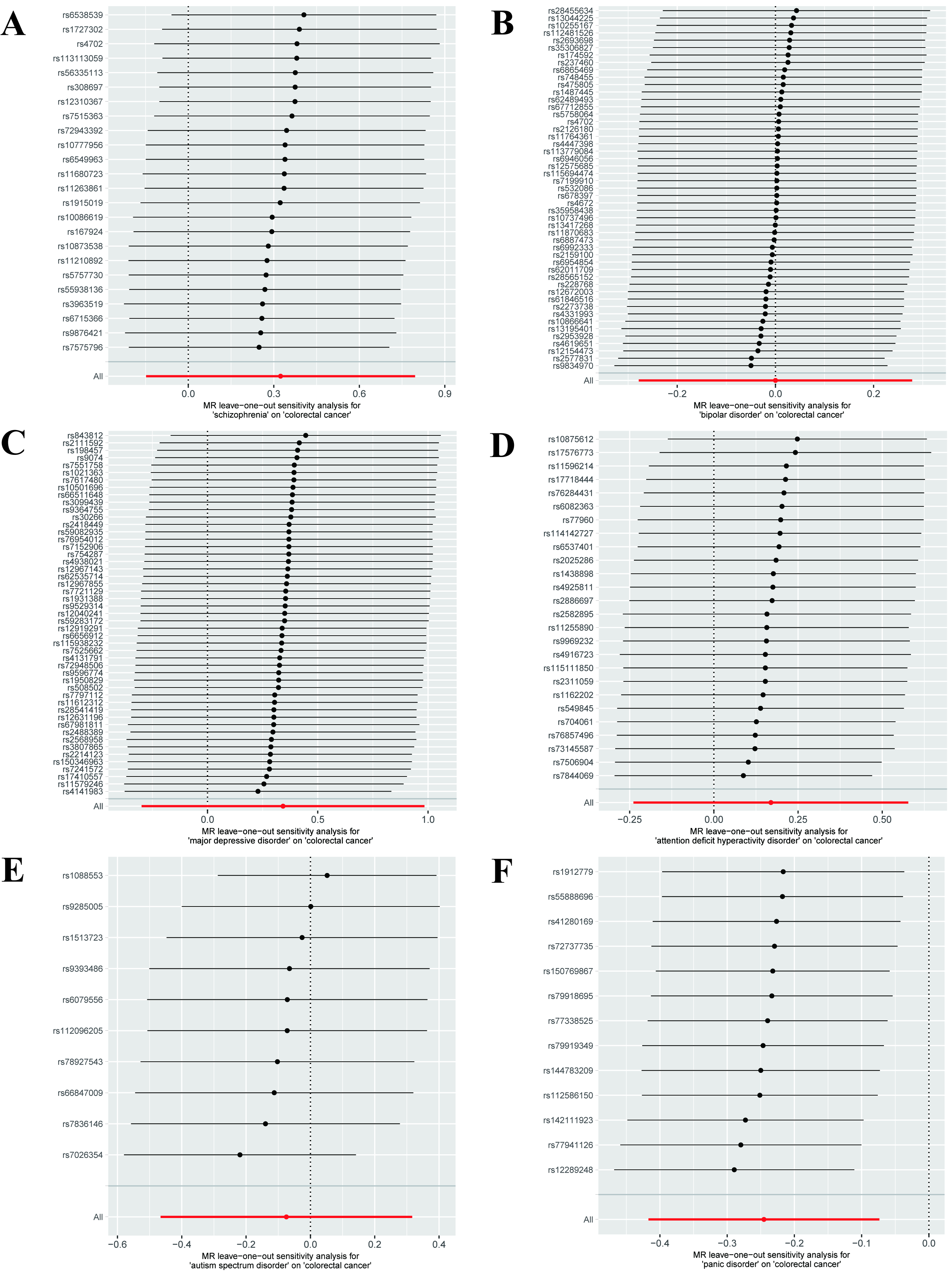

Supplement: Supplementary file 6 — Supplementary Figure 6. [file 41598_2024_66535_MOESM6_ESM.tif]
