# Supplementary material for: Causality between six psychiatric disorders and digestive tract cancers risk: a two-sample Mendelian randomization study
Source: Sci Rep. 2024 Jul 19;14:16689. doi: 10.1038/s41598-024-66535-7 (PMC11271641; doi:10.1038/s41598-024-66535-7)
Supplement: Supplementary file 9 — Supplementary Table 3. [file 41598_2024_66535_MOESM9_ESM.docx]

**Table S3.** Heterogeneity of MR analysis for mental illness and EC risk

| **Exposure** | **Outcome** | **Method** | **Q** | **Q_df** | **Q_*P* value** |
| --- | --- | --- | --- | --- | --- |
| Schizophrenia | EC | IVW | 29.85 | 23 | 0.15 |
|  |  | MR-Egger | 29.45 | 22 | 0.13 |
| BD | EC | IVW | 40.69 | 48 | 0.76 |
|  |  | MR-Egger | 40.54 | 47 | 0.74 |
| MDD | EC | IVW | 37.30 | 48 | 0.87 |
|  |  | MR-Egger | 36.75 | 47 | 0.86 |
| ADHD | EC | IVW | 23.16 | 25 | 0.57 |
|  |  | MR-Egger | 20.31 | 24 | 0.68 |
| ASD | EC | IVW | 7.05 | 9 | 0.63 |
|  |  | MR-Egger | 6.91 | 8 | 0.55 |
| PD | EC | IVW | 13.91 | 12 | 0.31 |
|  |  | MR-Egger | 11.21 | 11 | 0.43 |

EC, [Esophagus Cancer](javascript:;); BD, Bipolar Disorder; MDD, Major Depressive Disorder; ADHD, Attention Deficit Hyperactivity Disorder; ASD, Autism Spectrum Disorder; PD, Panic Disorder; IVW, Inversevariance Weighted
