# Supplementary material for: Causality between six psychiatric disorders and digestive tract cancers risk: a two-sample Mendelian randomization study
Source: Sci Rep. 2024 Jul 19;14:16689. doi: 10.1038/s41598-024-66535-7 (PMC11271641; doi:10.1038/s41598-024-66535-7)
Supplement: Supplementary file 10 — Supplementary Table 4. [file 41598_2024_66535_MOESM10_ESM.docx]

**Table S4.** The results of MR-Egger intercept analysis for mental illness and EC risk

| **Exposure** | **Outcome** | **MR-Egger intercept** | **SE** | ***P*** |
| --- | --- | --- | --- | --- |
| Schizophrenia | EC | 0.08 | 0.15 | 0.59 |
| BD | EC | 0.03 | 0.08 | 0.71 |
| MDD | EC | 0.06 | 0.09 | 0.46 |
| ADHD | EC | 0.16 | 0.10 | 0.10 |
| ASD | EC | 0.05 | 0.15 | 0.72 |
| PD | EC | -0.24 | 0.15 | 0.13 |

EC, [esophagus cancer](javascript:;); BD, Bipolar Disorder; MDD, Major Depressive Disorder; ADHD, Attention

Deficit Hyperactivity Disorder; ASD, Autism Spectrum Disorder; PD,Panic Disorder
