# Supplementary material for: Causality between six psychiatric disorders and digestive tract cancers risk: a two-sample Mendelian randomization study
Source: Sci Rep. 2024 Jul 19;14:16689. doi: 10.1038/s41598-024-66535-7 (PMC11271641; doi:10.1038/s41598-024-66535-7)
Supplement: Supplementary file 11 — Supplementary Table 5. [file 41598_2024_66535_MOESM11_ESM.docx]

**Table S5.** Heterogeneity of MR analysis for mental illness and GC risk

| **Exposure** | **Outcome** | **Method** | **Q** | **Q_df** | **Q_*P* value** |
| --- | --- | --- | --- | --- | --- |
| Schizophrenia | GC | IVW | 20.77 | 23 | 0.60 |
|  |  | MR-Egger | 19.28 | 22 | 0.63 |
| BD | GC | IVW | 50.29 | 48 | 0.38 |
|  |  | MR-Egger | 50.29 | 47 | 0.34 |
| MDD | GC | IVW | 58.14 | 48 | 0.15 |
|  |  | MR-Egger | 57.12 | 47 | 0.15 |
| ADHD | GC | IVW | 22.03 | 25 | 0.63 |
|  |  | MR-Egger | 20.96 | 24 | 0.64 |
| ASD | GC | IVW | 5.67 | 9 | 0.77 |
|  |  | MR-Egger | 5.52 | 8 | 0.70 |
| PD | GC | IVW | 19.48 | 12 | 0.08 |
|  |  | MR-Egger | 19.29 | 11 | 0.06 |

GC, [Gastric Cancer](javascript:;); BD, Bipolar Disorder; MDD, Major Depressive Disorder; ADHD, Attention Deficit Hyperactivity Disorder; ASD, Autism Spectrum Disorder; PD, Panic Disorder; IVW, Inversevariance Weighted
