# Supplementary material for: Causality between six psychiatric disorders and digestive tract cancers risk: a two-sample Mendelian randomization study
Source: Sci Rep. 2024 Jul 19;14:16689. doi: 10.1038/s41598-024-66535-7 (PMC11271641; doi:10.1038/s41598-024-66535-7)
Supplement: Supplementary file 12 — Supplementary Table 6. [file 41598_2024_66535_MOESM12_ESM.docx]

**Table S6.** The results of MR-Egger intercept analysis for mental illness and GC risk

| **Exposure** | **Outcome** | **MR-Egger intercept** | **SE** | ***P*** |
| --- | --- | --- | --- | --- |
| Schizophrenia | GC | -0.18 | 0.15 | 0.24 |
| BD | GC | -0.01 | 0.10 | 1.00 |
| MDD | GC | -0.10 | 0.11 | 0.36 |
| ADHD | GC | 0.12 | 0.11 | 0.31 |
| ASD | GC | 0.07 | 0.17 | 0.71 |
| PD | GC | -0.07 | 0.22 | 0.75 |

GC, [gastric cancer](javascript:;); BD, Bipolar Disorder; MDD, Major Depressive Disorder; ADHD, Attention

Deficit Hyperactivity Disorder; ASD, Autism Spectrum Disorder; PD, Panic Disorder
