# Supplementary material for: Causality between six psychiatric disorders and digestive tract cancers risk: a two-sample Mendelian randomization study
Source: Sci Rep. 2024 Jul 19;14:16689. doi: 10.1038/s41598-024-66535-7 (PMC11271641; doi:10.1038/s41598-024-66535-7)
Supplement: Supplementary file 13 — Supplementary Table 7. [file 41598_2024_66535_MOESM13_ESM.docx]

**Table S7.** Heterogeneity of MR analysis for mental illness and CRC risk

| **Exposure** | **Outcome** | **Method** | **Q** | **Q_df** | **Q_*P* value** |
| --- | --- | --- | --- | --- | --- |
| Schizophrenia | CRC | IVW | 31.51 | 23 | 0.11 |
|  |  | MR-Egger | 30.32 | 22 | 0.11 |
| BD | CRC | IVW | 52.28 | 48 | 0.31 |
|  |  | MR-Egger | 46.14 | 47 | 0.51 |
| MDD | CRC | IVW | 57.81 | 48 | 0.16 |
|  |  | MR-Egger | 57.27 | 47 | 0.15 |
| ADHD | CRC | IVW | 31.44 | 25 | 0.18 |
|  |  | MR-Egger | 31.39 | 24 | 0.15 |
| ASD | CRC | IVW | 19.84 | 9 | 0.02 |
|  |  | MR-Egger | 18.71 | 8 | 0.02 |
| PD | CRC | IVW | 10.15 | 12 | 0.60 |
|  |  | MR-Egger | 7.97 | 11 | 0.72 |

CRC, [Colorectal Cancer](javascript:;); BD, Bipolar Disorder; MDD, Major Depressive Disorder; ADHD, Attention Deficit Hyperactivity Disorder; ASD, Autism Spectrum Disorder; PD, Panic Disorder; IVW, Inversevariance Weighted
