# Supplementary material for: Causality between six psychiatric disorders and digestive tract cancers risk: a two-sample Mendelian randomization study
Source: Sci Rep. 2024 Jul 19;14:16689. doi: 10.1038/s41598-024-66535-7 (PMC11271641; doi:10.1038/s41598-024-66535-7)
Supplement: Supplementary file 14 — Supplementary Table 8. [file 41598_2024_66535_MOESM14_ESM.docx]

**Table S8.** The results of MR-Egger intercept analysis for mental illness and CRC risk

| **Exposure** | **Outcome** | **MR-Egger intercept** | **SE** | ***P*** |
| --- | --- | --- | --- | --- |
| Schizophrenia | CRC | -0.08 | 0.08 | 0.36 |
| BD | CRC | -0.11 | 0.04 | 0.17 |
| MDD | CRC | -0.04 | 0.05 | 0.51 |
| ADHD | CRC | 0.02 | 0.06 | 0.76 |
| ASD | CRC | -0.09 | 0.13 | 0.51 |
| PD | CRC | 0.12 | 0.08 | 0.17 |

CRC, [Colorectal cancer](javascript:;); BD, Bipolar Disorder; MDD, Major Depressive Disorder; ADHD, Attention

Deficit Hyperactivity Disorder; ASD, Autism Spectrum Disorder; PD, Panic Disorder
