# Supplementary material for: Causality between six psychiatric disorders and digestive tract cancers risk: a two-sample Mendelian randomization study
Source: Sci Rep. 2024 Jul 19;14:16689. doi: 10.1038/s41598-024-66535-7 (PMC11271641; doi:10.1038/s41598-024-66535-7)
Supplement: Supplementary file 15 — Supplementary Legends. [file 41598_2024_66535_MOESM15_ESM.docx]

Figure S1. Forest plot of causal relationship between psychiatric disorders and [esophagus](javascript:;) cancer risk. (A) schizophrenia, (B) bipolar disorder, (C) major depressive disorder, (D) attention deficit hyperactivity disorder, (E) autism spectrum disorder, (F) panic disorder

Figure S2. Sensitivity analysis results of “leave-one-out” between psychiatric disorders and [esophagus](javascript:;) cancer risk. (A) schizophrenia, (B) bipolar disorder, (C) major depressive disorder, (D) attention deficit hyperactivity disorder, (E) autism spectrum disorder, (F) panic disorder. The bars indicate the CI. Circles indicate that if each SNP is missing, Mendelian randomization assessment is performed using the fixed effects model inverse variance weighted (IVW)

Figure S3. Forest plot of causal relationship between psychiatric disorders and gastric cancer risk.. (A) schizophrenia, (B) bipolar disorder, (C) major depressive disorder, (D) attention deficit hyperactivity disorder, (E) autism spectrum disorder, (F) panic disorder

Figure S4. Sensitivity analysis results of “leave-one-out” between psychiatric disorders and gastric cancer risk. (A) schizophrenia, (B) bipolar disorder, (C) major depressive disorder, (D) attention deficit hyperactivity disorder, (E) autism spectrum disorder, (F) panic disorder. The bars indicate the CI. Circles indicate that if each SNP is missing, Mendelian randomization assessment is performed using the fixed effects model inverse variance weighted (IVW)

Figure S5. Forest plot of causal relationship between psychiatric disorders and colorectal cancer risk. (A) schizophrenia, (B) bipolar disorder, (C) major depressive disorder, (D) attention deficit hyperactivity disorder, (E) autism spectrum disorder, (F) panic disorder.

Figure S6. Sensitivity analysis results of “leave-one-out” between psychiatric disorders and colorectal cancer risk. (A) schizophrenia, (B) bipolar disorder, (C) major depressive disorder, (D) attention deficit hyperactivity disorder, (E) autism spectrum disorder, (F) panic disorder. The bars indicate the CI. Circles indicate that if each SNP is missing, Mendelian randomization assessment is performed using the fixed effects model inverse variance weighted (IVW)
